# An evolutionary ratchet leading to loss of elongation factors in eukaryotes

**DOI:** 10.1186/1471-2148-14-35

**Published:** 2014-02-24

**Authors:** Gemma C Atkinson, Anton Kuzmenko, Ivan Chicherin, Axel Soosaar, Tanel Tenson, Martin Carr, Piotr Kamenski, Vasili Hauryliuk

**Affiliations:** 1 University of Tartu, Institute of Technology, Nooruse 1, 50411 Tartu, Estonia; 2 Department of Molecular Biology, Umeå University, Umeå, Sweden; 3 Laboratory for Molecular Infection Medicine Sweden (MIMS), Umeå University, Umeå, Sweden; 4 Department of Molecular Biology, Faculty of Biology, Moscow State University, Moscow, Russia; 5 School of Applied Sciences, University of Huddersfield, Queensgate, Huddersfield HD1 3DH, UK

**Keywords:** eEF1A, EFL, eEF1B, Ribosome, Elongation factor, GTPase, GEF, Molecular evolution, Eukaryotes

## Abstract

**Background:**

The GTPase eEF1A is the eukaryotic factor responsible for the essential, universal function of aminoacyl-tRNA delivery to the ribosome. Surprisingly, eEF1A is not universally present in eukaryotes, being replaced by the paralog EFL independently in multiple lineages. The driving force behind this unusually frequent replacement is poorly understood.

**Results:**

Through sequence searching of genomic and EST databases, we find a striking association of eEF1A replacement by EFL and loss of eEF1A’s guanine exchange factor, eEF1Bα, suggesting that EFL is able to spontaneously recharge with GTP. Sequence conservation and homology modeling analyses indicate several sequence regions that may be responsible for EFL’s lack of requirement for eEF1Bα.

**Conclusions:**

We propose that the unusual pattern of eEF1A, eEF1Bα and EFL presence and absence can be explained by a ratchet-like process: if either eEF1A or eEF1Bα diverges beyond functionality in the presence of EFL, the system is unable to return to the ancestral, eEF1A:eEFBα-driven state.

## Background

EF1A in eukaryotes (eEF1A) and archaea (aEF1A) is a highly expressed essential GTPase translation factor. Just like its bacterial ortholog, EF-Tu, EF1A delivers aminoacyl-tRNA (aa-tRNA) to the ribosome in complex with GTP during the elongation stage of translation. Accommodation of the aa-tRNA in the ribosomal A site induces GTP hydrolysis by EF1A, releasing the GDP-bound factor from the ribosome [1]. GDP bound to eEF1A needs to be replaced with GTP for the next functional cycle to begin. Some translational GTPases such as the close relative of EF1A, SelB, dissociate GDP rapidly, which leads to spontaneous recharging [2]. However, dissociation of GDP from EF-Tu and EF1A is extremely slow [3,4] and therefore these GTPases require a dedicated guanine exchange factor (GEF) for recharging: EF-Ts in bacteria and EF1B in eukaryotes (eEF1B) and archaea (aEF1B) [5]. Unlike EF-Ts, eEF1B is a multi-subunit protein, with GEF activity residing in the alpha subunit (eEF1Bα) [3,6]. The crystal structure of the eEF1A:eEF1Bα carboxy terminus complex has shed light on the mechanisms of exchange at the molecular level, showing which parts of eEF1A and eEF1Bα interact and how this brings about GDP dissociation [7,8].

Besides its role in translation, eEF1A has a variety of additional, “moonlighting” functions. These include actin bundling, nuclear export of aa-tRNAs, proteolysis of misfolded proteins, modulating apoptosis, response to amino acid starvation and viral replication [9,10]. Being a universally essential protein in translation, and an accessory protein in a variety of other processes, the discovery of a lack of eEF1A in some eukaryotes was unexpected [11]. In these eEF1A-lacking organisms, another related factor, EFL (for EF1A-like), is present. EFL carries the same domain structure as eEF1A and is presumably functionally equivalent. Most surprising is the broad but discontinuous distribution of EFL in eukaryotes and usually mutual exclusivity with eEF1A [11,12]. The pattern of presence and absence has been explained both by horizontal gene transfer (HGT) and co-maintenance and long term co-maintenance followed by lineage sorting (some lineages losing eEF1A and some losing EFL) [12-17]. Given the absence of strong support for any specific instance of HGT of EFL, the possibility that the last common ancestor of eukaryotes carried both eEF1A and EFL can not be ruled out [12]. However, this would have required millions of years of functional redundancy in the co-maintenance of eEF1A and EFL without either being lost before the divergence of all eukaryotic groups that carry EFL. Regardless of the mode of descent of EFL, the ancestral state at the last common ancestor of eukaryotes and archaea is likely to be co-presence of eEF1A and eEF1Bα and absence of EFL, as aEF1A and aEF1B are found in all archaea, but EFL has never been identified in this domain of life.

Due to a near complete absence of experimental investigations of EFL, the evolutionary mechanisms driving mutual exclusivity of eEF1A and EFL are poorly understood. We hypothesize that the key to this phenomenon lies in the differences in the functional cycle of the two proteins. It was briefly noted [15] that the GEF eEF1Bα has not been identified in the genomes of EFL-containing organisms *Thalassiosira*, *Chlamydomonas* and *Ostreococcus*, suggesting that EFL may self-recharge like some other translational GTPases, or that a non-homologous GEF may be involved. With the increasing number of genomes and large scale EST data available for many eukaryotes including those that carry EFL, we have conducted a large-scale survey of EFL, eEF1A and eEF1Bα presence and absence across the eukaryotic tree of life. We show a striking association of EFL presence with loss of eEF1Bα and eEF1A. We hypothesise a ratchet-like evolutionary process of reduction: if eEF1A or eEF1Bα diverges beyond functionality in the presence of EFL, the system is unable to return to the ancestral, eEF1A:eEFBα-driven state. Whether EFL loss is similarly irremediable depends on the rate of HGT of this factor.

Motivated by our hypothesis, we set out to test whether EFL can substitute for a loss of eEF1Bα in an organism possessing eEF1A gene. Similarly to a previous study that found *Diplomena* EFL can not substitute for eEF1A in *Trypanosoma*[18]*,* we find that *Monosiga brevicolis* EFL is unable to substitute for either double eEF1A and eEF1Bα or single eEF1Bα deletion in *Saccharomyces cerevisiae*, thus suggesting an existence of functional barriers to the spread of EFL across eukaryotes.

## Results

**Figure 1:**
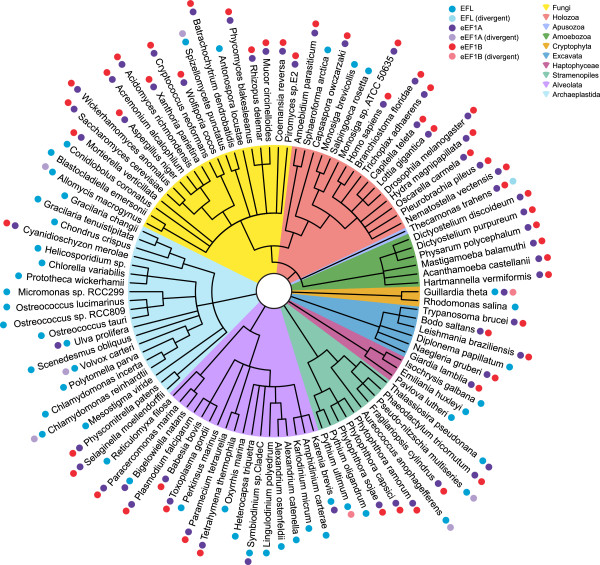
**Cladogram showing presence and absence of elongation factors across eukaryotes.** The tree summarizes current knowledge of the taxonomic grouping of the species considered here. Polytomies are present where branching order is unknown or contentious. Colored shading behind branches indicates major lineages, as per the color key in the top left. Circles show presence and absence of intact (opaque) or degraded (semi-transparent) elongation factors, with colors indicating factor identity according to the top left key.

Sequence searching of genomic and EST databases shows a striking pattern of presence and absence of EFL, eEF1A and eEF1Bα (Figure 1 and Additional file 1: Table S1). Both EFL and eEF1A are present in all major lineages of eukaryotes, and their presence is mostly – but not universally – mutually exclusive, as previously reported [11]. Supporting the result of Szabova *et al.*[18] that experimentally showed eEF1A and EFL can be co-maintained without affecting growth*,* eEF1A and EFL can be co-maintained in a modest number of organisms (*Thalassiosira pseudonana*, *Guillardia theta, Karenia brevis, Symbiodinium* sp. and *Ulva prolifera*), although these organisms carry no detectable eEF1Bα. The gut fungus *Basidiobolus ranarum* has also been found to carry both eEF1A and EFL [19], however in the absence of a genome or EST project to search for eEF1Bα, it was not included in our survey. This co-maintenance may reflect incomplete lineage sorting, or may be because eEF1A is still required for one of its moonlighting functions in these organisms. Importantly, we find the loss of eEF1A is almost universally accompanied by parallel loss of eEF1Bα, suggesting that EFL functioning does not require the assistance of this GEF (Figure 1 and Additional file 1: Table S1). In some rare cases where eEF1A or eEF1Bα are detectable along with EFL, degradation of eEF1A or eEF1Bα sequence is apparent, even in functionally important sites; eEF1A appears to be evolving with an apparent loss of selective constraint on the protein sequence in *Allomyces macrogynus, Aspergillus niger, Pseudo-nitzschia multiseries, Fragilariopsis cylindrus, Bigelowiella natans, Chlamydomonas reinhardtii* and *Volvox carteri*, while eEF1Bα is in the process of decay in *Guillardia theta* and *Pythium ultimum. Thecamonas trahens* on the other hand encodes a divergent EFL along with eEF1A and an apparent eEF1B-kinase protein fusion (Figure 1 and Additional file 1: Table S1). Thus, in our snapshot of eukaryotic history, we have captured multiple ongoing cases of gene degradation towards loss. Overall, the association of EFL presence with eEF1A and eEF1Bα loss is statistically significant whether the divergent sequences are considered as present or absent (*p* <0.00001 in both cases).

Phylogenetic analysis of the EFL sequences detected in this study gives a tree that is overall similar to other published EFL phylogenies (for example [13,14,16,20]), although with fewer taxa as we only considered organisms with large scale EST and whole genome data available (Additional file 2: Figure S1A). Even with additional sequences from PCR amplification and sequencing of individual genes, phylogenetic analysis of EFL does not shed much light on the origin and deep evolutionary history of EFL; the deepest branches in the phylogenetic tree lack strong statistical support in ML and/or Bayesian analyses, and the EFL tree can not be rooted reliably due to long branch attraction of divergent sequences to the outgroup [20]. Therefore, the path to EFL replacement of eEF1A (HGT or co-maintenance with differential loss) is hard to determine, as also found with probabilistic models of EFL gain and loss [21]. However, we do see strong bootstrap and posterior probability support for some taxonomic assemblages within the EFL tree, for example Dinophyceae (89% MLBP (maximum likelihood bootstrap percentage) and 1.0 BIPP (Bayesian inference posterior probability)), Rhodophyta (99% MLBP, 1.0 BIPP), fungi, excluding *Conidiobolus cornatus* (99% MLBP, 1.0 BIPP), stramenopiles (Heterokontophyta + Oomycetes, 100% MLBP, 1.0 BIPP) and members of Choanoflagellatea + Ichthyosporea (100% MLBP, 1.0 BIPP). Dinophyceae and Rhodophyta are all EFL encoding, suggesting that EFL was vertically inherited within these groups. Surprisingly, EST evidence suggests eEF1A has not been completely lost in Dinophyceae, with eEF1A ESTs being detected in *Symbiodinium* sp. and *Karenina brevis* (Figure 1 and Additional file 1: Table S1). In the case of Choanoflagellatea, Ichthyosporea, stramenopiles and fungi, some species encode EFL while some encode eEF1A. It is unclear whether co-maintenance and differential loss alone is responsible for this distribution, or whether HGT has been involved. Whatever the source of EFL in these taxa, lineage sorting appears incomplete, with eEF1A sequence relics being detected in some EFL-encoding stramenopiles and fungi. The backbone of the eEF1A tree is poorly resolved and multiple paralogs of eEF1A are apparent (Additional file 2: Figure S1B). Some of these are highly divergent, such as those from *Tetrahymena thermophila* and *Paramecium tetraurelia*, which are attracted to the degrading eEF1As in organisms encoding EFL (Additional file 2: Figure S1B).

**Figure 2:**
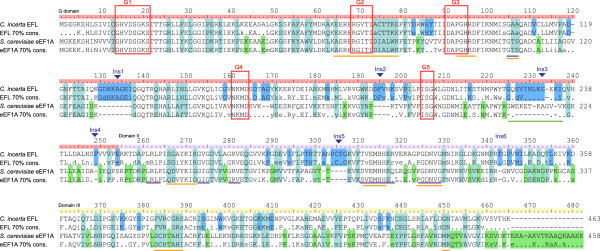
**Consensus and example sequence alignment of eEF1A and EFL.** Consensus sequences aligned with example sequences for eEF1A and EFL were calculated at the 70% level using the Python program Consensus Finder [34]. Ruler coloring indicates the boundaries of the three domains. Shading behind residues shows conservation patterns: turquoise – strongly differentially conserved sites; blue – sites conserved in EFL but not eEF1A; green – sites conserved in eEF1A and not in EFL. Red boxes indicate the location of the nucleotide binding motifs of the G domain. Colored lines beneath the alignment indicate structural features as follows: orange – eEF1Bα interacting sites; blue – residues lining the amino-acyl moiety binding pocket; green – the extended loop of the helix-loop-helix on the ribosome binding surface.

Comparison of patterns of evolution across sites using a consensus sequence alignment of eEF1A versus EFL (Figure 2) shows differentially conserved sites across all domains (G domain, domain II and domain III), with some such sites clustering together. To determine how the sequence changes affect the structural contacts of EFL, a homology model was made of *Chlamydomonas incerta* EFL using the X-ray crystal structure of *S. cerevisiae* eEF1A in complex with eEF1Bα as the template (PDB ID 1IJE) [8].

**Figure 3:**
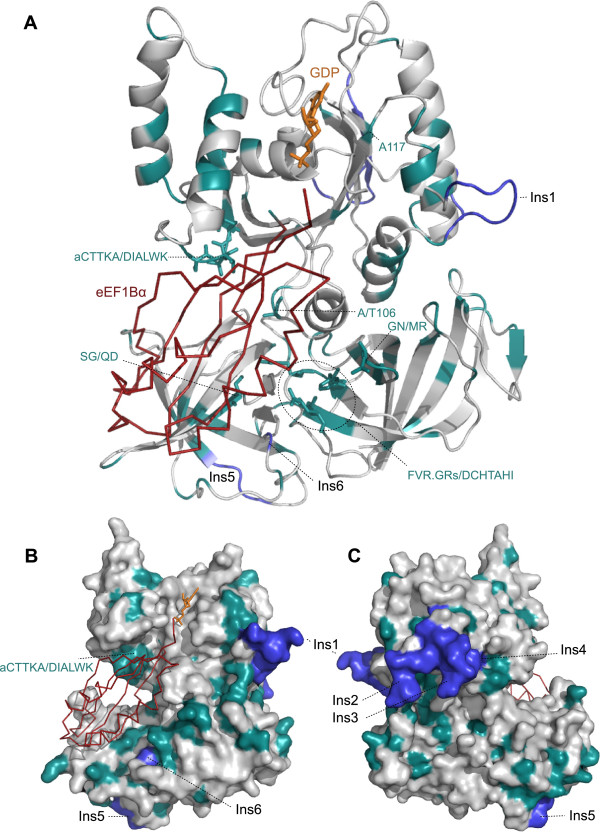
**Homology model of EFL in complex with eEF1Bα.** The homology model on EFL based on the structure of eEF1A in complex with eEF1Bα (red ribbon) is shown **A)** in cartoon form, **B)** as a surface, showing the exposed face when in complex with the ribosome, and **C)** as a surface showing the ribosome binding face. In all panels, turquoise coloring shows strongly differentially conserved sites as per the alignment in Figure 2, and blue parts of the structure show conserved insertions in EFL relative to eEF1A. Residues shown as turquoise sticks are those differentially conserved sites that in eEF1A interact with eEF1Bα.

Many of the eEF1A residues that interact with eEF1Bα overlap with regions important for nucleotide or aa-tRNA binding (Figure 2). Therefore, it is unsurprising that these multifunctional regions are well conserved in EFL. However, regions of eEF1A that are apparently specialized for eEF1Bα binding are often very different in EFL. The GTPase (G) domain is overall well conserved, particularly in the nucleotide binding loops, with less conservation seen in the exposed loops in between. One of the most striking differences between eEF1A and EFL in this domain is a six amino acid-long strongly differentially conserved patch, Figure 2 coordinates 74-79, (consensus sequences aCTTKA in EFL and DIALWK in eEF1A) that is located in a helix between the G2 and G3 nucleotide binding motifs. The DIALWK motif is part of a loop of eEF1A that directly interacts with eEF1Bα through hydrogen bonds (Figure 3A and B) [7]. The conformation of this loop is also stabilized by another strongly differentially conserved site: alignment position 35 (D35 in eEF1A and P35 in EFL, Figure 2). This suggests that the striking sequence differences in these residues are directly related to EFL’s apparent lack of requirement for eEF1Bα. Another differentially conserved residue of this domain that in eEF1A may interact with eEF1Bα is in position 106 in Figure 2 (T106 in eEF1A and A106 in EFL, Figure 3A).

Three of the five sequence insertions in EFL relative to eEF1A (Ins1-5, Figure 2) are found in the G domain. Structural alignment of EFL with the structure of eEF1A’s bacterial ortholog EF-Tu on the ribosome [22] suggests Ins1 and Ins2 are exposed with no obvious ribosomal interaction partners, but Ins3 extends a helix-loop-helix structural element on the ribosome-binding face (Figure 3C). This insertion is also interesting as it overlaps with a 12 amino acid insertion in opisthokont eEF1A [23]. However, the sequence alignment of these two insertions relative to each other is ambiguous, and thus there is no evidence that the Ins3 insertion is homologous to the animal/fungal insertion. A single conserved amino acid deletion in EFL relative to eEF1A is also apparent, but is found in an exposed loop of eEF1A that is poorly conserved (position 120 in Figure 2).

In domain II, very strong conservation between eEF1A and EFL is seen in the regions that in bacterial EF-Tu form the pocket for accommodating the aminoacyl moiety of aa-tRNA [22] (Figure 2). Differentially conserved sites in this domain are largely in exposed loops, however there are three residues that in eEF1A are positioned to potentially interact with eEF1Bα and are strongly conserved as chemically different amino acids in EFL: alignment coordinates 266-267 and 272, corresponding to Q249, D250 and G255 in eEF1A; respectively S264, G265, K270 in EFL (Figures 2 and 3A).

In domain III, differentially conserved sites are mostly dispersed and largely exposed. However, two differentially conserved regions are positioned to be involved in the eEF1A:eEF1Bα interaction (Figures 2 and 3A): firstly DCHTAHI in eEF1A, which is FVR.GRs in EFL (starting at position 383 in Figure 2, 360 in *S. cerevisiae* eEF1A and 381 in *C. incerta* EFL), and secondly amino acids GN in EFL, conserved as MR in eEF1A starting at position 450 in Figure 2, 427 in *S. cerevisiae* eEF1A and 448 in *C. incerta* EFL). eEF1A is extended in sequence at the extreme C terminus by an average of 17 amino acids, relative to EFL (Figure 2). The amino acid contacts in this region are unknown as they are not present in the crystal structure.

Mutants of eEF1A in *S. cerevisiae* have previously been shown to confer independence from eEF1Bα: R164K, T22S, A112T, and A117V [24] (Figure 2). These are mostly located in the G domain and surprisingly, only two differ in conservation between eEF1A and EFL: A112 is unconserved in EFL, and A177 is differentially conserved as P117 in EFL. This suggests there are multiple routes to independence from eEF1Bα, although the single amino acid replacement routes may be highly species-specific in their effectiveness. There have been no cases of natural eEF1Bα-free eEF1As reported, and our distribution analysis suggests this would be rare.

Biochemical experimentation demonstrating rapid self-recharging of EFL:GDP with GTP would be the unequivocal proof of our hypothesis that EFL can functionally substitute for eEF1A and eEF1Bα loss. However, all our attempts to overexpress EFL in *E. coli* have failed (data not shown). An alternative strategy is to perform *in vivo* complementation experiments by replacing either both eEF1A and eEF1Bα or just eEF1Bα with EFL. Using *S. cerevisiae* as a model organism, we generated strains with controlled expression of eEF1A, eEF1Bα and *M. brevicollis* EFL (see Additional file 3: Materials and methods). Removal of saver plasmids expressing either both eEF1Bα- or eEF1A or just eEF1Bα by addition of 5-Fluoroorotic acid (5-FoA) resulted in loss of viability that was not rescued by expression of *M. brevicollis* EFL (Additional file 4: Figure S2), indicating that in *S. cerevisiae*, EFL does not seem to be able to complement the loss of either eEF1Bα alone or eEF1Bα and both genes encoding eEF1A in *S. cerevisiae* (TEF1 and TEF2 [25]).

## Discussion

**Figure 4:**
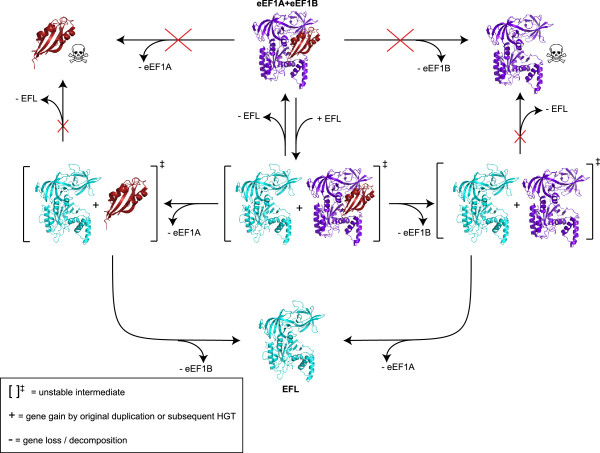
**Evolutionary dynamics of elongation factors.** The model shows the possible and impossible combinations of elongation factors EFL (turquoise) eEF1A (purple), eEF1Bα (red) following gene acquisition and loss. Impossible scenarios (those that would be fatal for the organism) are indicated with a skull and crossbones. Other notation is explained in the inset box.

The pattern of presence and absence of EFL, eEF1A and eEF1Bα allows us to derive a ratchet-like model for the evolutionary dynamics of elongation factor gain and loss in terms of the viable and inviable fates of different combinations of elongation factors (Figure 4). It is likely is that EFL arose by gene duplication after the last common ancestor of eukaryotes and archaea, which encoded eEF1A and eEF1Bα. Degradation of eEF1A or eEF1Bα though random genetic drift in the presence of EFL is likely to be an almost irreversible step that acts as the pawl of the ratchet: a return to the ancestral state would require that the lost gene is quickly re-transferred before its binding partner diverges beyond preventing functional interaction: an unlikely scenario.

The ratchet may also work in the reverse direction, i.e. once EFL is lost from an EFL + eEF1A + eEF1Bα-encoding organism there is no going back. This depends on how frequently, if at all, EFL is transmitted by HGT, which is currently unclear [12-17]. In the absence of EFL HGT, the ratchet is nonprocessive and acts merely as a lineage-sorting evolutionary mechanism, degrading EFL + eEF1A + eEF1Bα-encoding organisms into either EFL- or eEF1A + eEF1Bα-encoding. Repetitive re-introduction of EFL by HGT into an eEF1A + eEF1Bα background would make the ratchet processive, potentially leading to an enrichment of EFL-containing organisms. Given the uncertainty in EFL HGT rates, it is impossible to assess the processivity of the ratchet. In the most extreme case of nonprocessivity, EFL would not have been subject to HGT at all, would have been present in the eukaryotic ancestor, and then all three genes would have been maintained for millions of years before the divergence of all modern EFL-encoding groups of organisms.

The relative rates of transitioning between states of factor composition is likely to be highly species specific; while EFL is clearly capable of replacing eEF1A in multiple lineages, it has been experimentally shown that *Diplonema* EFL can be co-maintained with, but can not replace eEF1A in *Trypanosoma*[18]. Our results suggest the same is true for replacement of either both eEF1A and eEF1Bα or just eEF1Bα with *M. brevicollis* EFL in *S. cerevisiae* (Additional file 4: Figure S2). EFL is not naturally found in any yeasts, which may reflect an irreplaceability of yeast eEF1A, or may be because successful HGT is rare in this group of organisms [26].

The stability of the intermediate states (EFL in combination with eEF1A and/or eEF1Bα) depends on organism-specific constraints such as multifunctionalisation (which may drive the system towards co-maintenance of both paralogs), and evolutionary selection for genome reduction (which could increase the rate of loss). The rare cases of dual maintenance may be driven by multifunctionality of eEF1A [12]. In fact it is surprising that eEF1A is not maintained in parallel to EFL more often given its plethora of “moonlighting functions”. One explanation could be that eEF1A is not universally multifunctional, or its additional functions do not provide enough selective advantage for its maintenance. It is also possible that some of the moonlighting functions could be carried out by EFL, or by one of the other two closely related paralogs of eEF1A, eRF3 or Hbs1p. Indeed, eRF3 and Hbs1p have already taken over eEF1A’s additional ancestral functions in translation termination and mRNA decay via eRF1 and Dom34p binding [27,28].

Our sequence conservation and homology modeling analyses indicate several sequence regions that may be responsible for EFL’s lack of requirement for eEF1Bα. However, we can not rule out the possibility that EFL has hijacked another GEF for recharging. One candidate could be eIF2α, the GEF for an ancient paralog of eEF1A and EFL, eIF2χ. Although homology is not apparent at the sequence level, eIF2α is structurally similar to eEF1Bα [29].

The ratchet mechanism of elongation factor replacement relies only on random genetic drift and can explain how eEF1A can be efficiently replaced by EFL without the need of the latter being a “better” elongation factor, i.e. providing a selective advantage in itself. The ability of EFL to recharge without a specialized accessory factor does not in itself make it an improved enzyme; the impact of this reduction on the functional cycle of the elongation factor is unknown.

## Conclusions

The genomic distribution of the guanine exchange factor eEF1Bα considered alongside that of EFL and eEF1A gives a very strong indication that EFL is able to recharge without this exchange factor. Thus, the presence of EFL has apparently allowed the decay and loss of both eEF1A and eEF1Bα in some lineages of eukaryotes, a ratchet-like process where return to the ancestral state is unlikely. Horizontal transmission of EFL has been proposed among eukaryotes, however current sequence data is inadequate for determining the rate of transfer, and indeed if it occurs at all. Additional sequencing efforts are required to more fully resolve the dynamics of EFL through the evolutionary history of eukaryotes. Further in vitro and in vivo experimentation is also required to answer the question of whether EFL self-recharges or whether exchange is promoted by another factor.

## Methods

BLAST searches were carried out at the JGI (http://genome.jgi.doe.gov/), NCBI (http://ncbi.nlm.nih.gov/), Origin of Multicellularity [30], GeneDB [31] and *Cyanidioschyzon merolae* genome (http://merolae.biol.s.u-tokyo.ac.jp/) database webpages, using *M. brevicolis* EFL, *Monosiga* sp. (ATCC 50635) eEF1A and *S. cerevisiae* eEF1Bα as queries. The BLASTp method was used where protein models were available; otherwise tBLASTn was used to search protein against translated genomic and EST nucleotide sequences. Nucleotide hits were translated into protein using Transeq at the EBI (http://www.ebi.ac.uk/Tools/). The E value limit was set to 1e-5 and sequences found with eEF1Bα were checked with Pfam to confirm identity based on the presence of the EF1_GNE (EF1 guanine nucleotide exchange) domain [32]. eEF1Bα has been subject to gene duplication in some lineages resulting in paralogs such as eEF1Bδ in Metazoa and eEF1Bβ in plants. As we are interested in presence or absence of a detectable eEF1Bα homolog and not the complete family tree of this protein family, which has been addressed elsewhere [5], only the top hit was retained. In house translational GTPase datasets (GCA) were used for classification of EFL and eEF1A sequences.

Sequences were aligned using MAFFT v6.864b with the L-ins-i strategy [33]. Consensus sequences were generated with the Consensus Finder Python script [34]. Only full length, non-degrading sequences or identical/nearly identical duplicates from the same organism were included in the data set. The threshold conservation level was set to 70%.

For phylogenetic analyses of eEF1A and EFL, gap-rich ambiguous alignment regions were identified by eye and removed. Extremely truncated sequences typical of ESTs were removed to minimize the amount of missing data. This resulted in dataset dimensions of 462 aligned amino acid positions from 72 sequences for EFL, and 446 positions from 82 sequences for eEF1A. Phylogenetic analyses were carried out with RAxML [35] and MrBayes [36] on the CIPRES Science Gateway v3.2 [37]. MrBayes was run with a mixed model plus the gamma rate distribution, with the program converging on the WAG model (1.0 posterior probability) in the case of EFL, and RTREV (0.99 posterior probability) in the case of eEF1A. Two independent runs of 4 chains were run for 2 million generations, sampling every 1000 generations. A consensus tree was generated after a burn in of 200000 generations. At the end of the runs, the standard deviations of split frequencies (SDSF) were 0.01 in the case of EFL and 0.1 in the case of eEF1A. RAxML was run taking into account the MrBayes model selection, with the WAG + CAT model for EFL and RTREV + CAT model for eEF1A with 100 bootstrap replicates in each case.

A structural homology model of EFL was generated using Swiss-Model [38] with the crystal structure of the eEF1A:eEF1Bα complex (PDB ID 1IJE, [8]) as the structural template. Using MacPyMOL (http://www.pymol.org), the EFL model was structurally aligned with the crystal structure of EF-Tu on the ribosome (PDB IDs 2WRN and 2WRO) [22] in order to indicate likely ribosome and aa-tRNA binding surfaces.

## Competing interests

The authors declare that they have no competing interests.

## Authors’ contributions

GCA and MC conceived the bioinformatic part of the project, and performed analyses. AK, PK and VH conceived and designed experiments, AK, AS, IC and PK performed experiments, GCA and VH wrote the paper with contributions from MC, AK, PK, and AS. TT and VH contributed reagents. All authors read and approved the final manuscript.

## Supplementary Material

Additional file 1: Table S1 Presence and absence of EFL eEF1A, eEF1Bα with sequence ID numbers. Sequences are ordered by taxonomy. Paler colours indicate that a sequence is present, but highly divergent. Click here for file

Additional file 2: Figure S1 Phylogenies of eEF1A and EFL. See Additional file 3 for legend. Click here for file

Additional file 3: Supplementary Methods, full legends for figures S1 and S2, and Table S2 of strains and plasmids used in the study. Click here for file

Additional file 4: Figure S2 Both *S. cerevisiae* double eEF1Bα and eEF1A knock-out and eEF1Bα single knock-out are not complemented *in vivo* by Monosiga brevicollis EFL. See Additional file 3 for legend. Click here for file
