# Supplementary figures and images for: An evolutionary ratchet leading to loss of elongation factors in eukaryotes
Source: BMC Evol Biol. 2014 Feb 24;14:35. doi: 10.1186/1471-2148-14-35 (PMC3938643; doi:10.1186/1471-2148-14-35)

Additional file 2 Figure S1

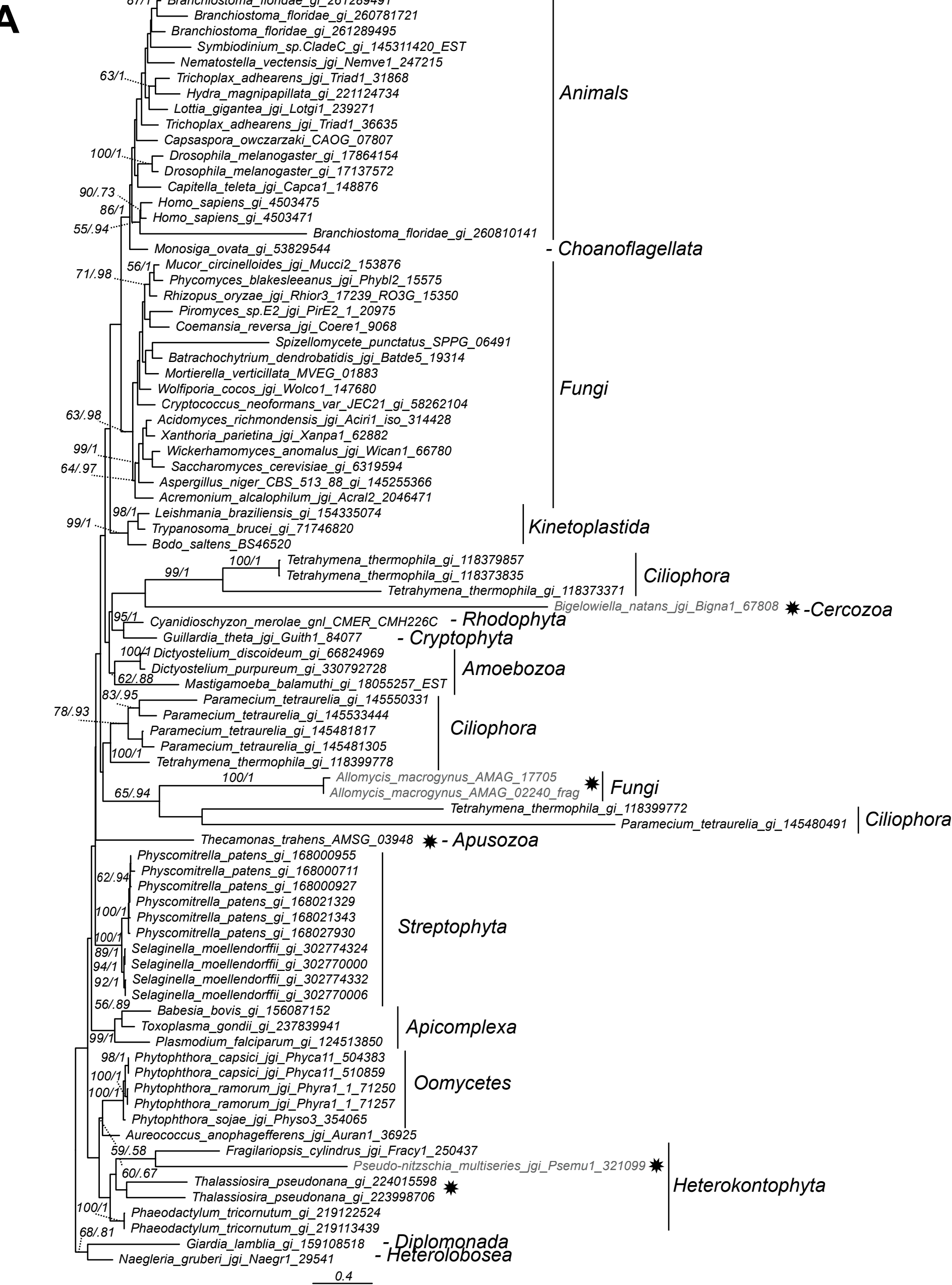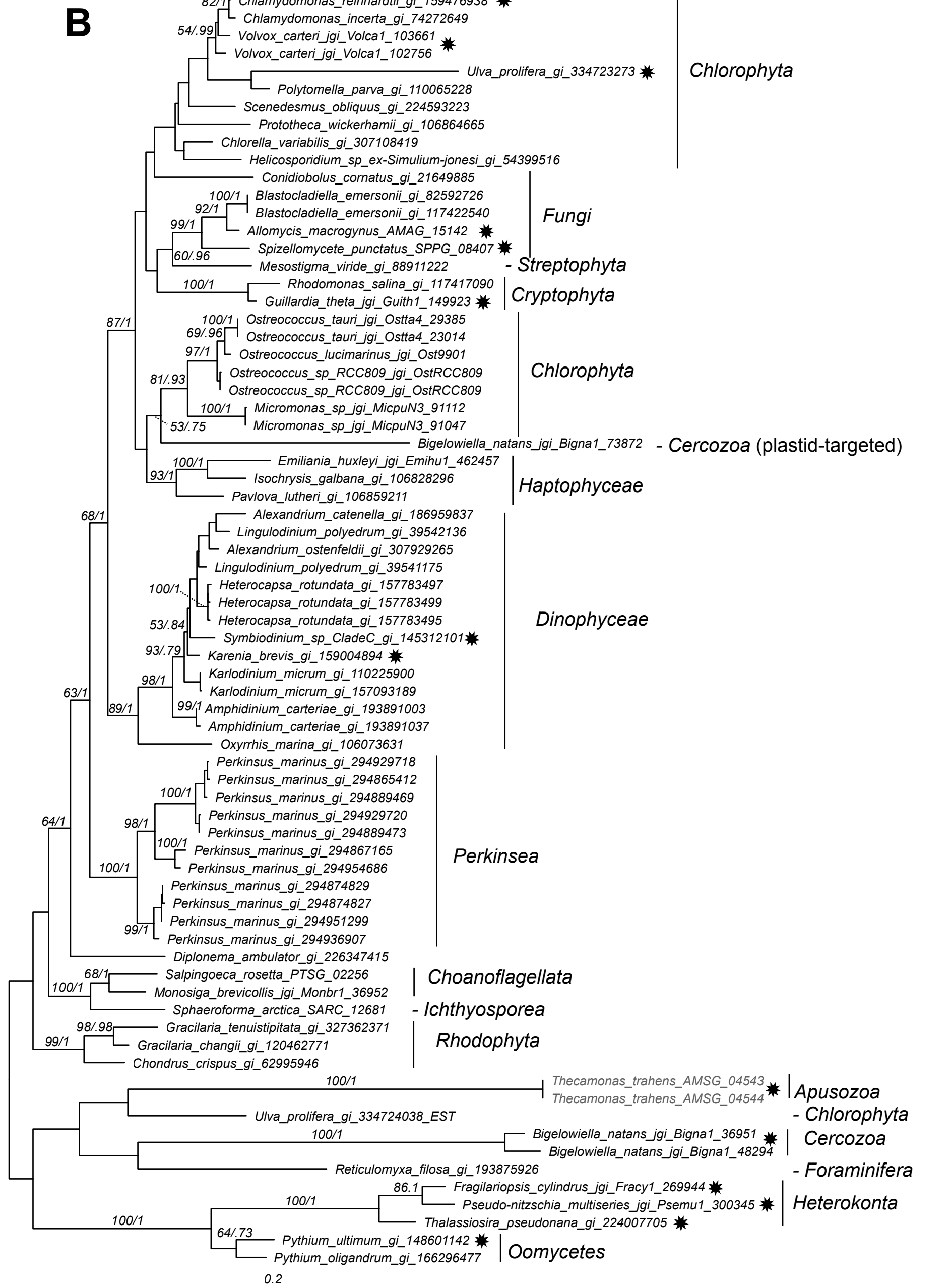

Supplement: Additional file 2: Figure S1 — Phylogenies of eEF1A and EFL. See Additional file 3 for legend. [file 1471-2148-14-35-S2.pdf]

**A**                      TEF1 EFL

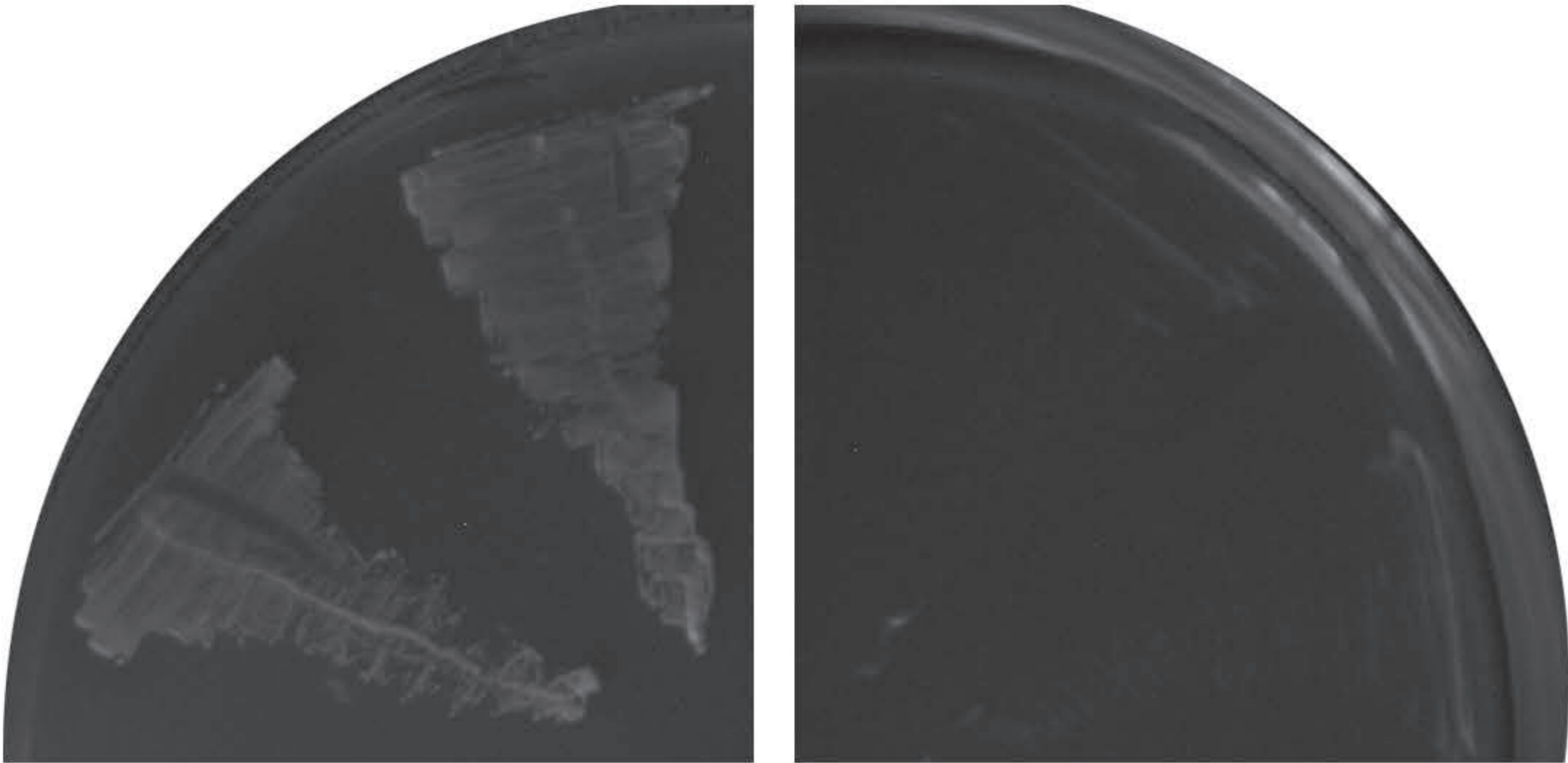

**B**                      TEF1 EFL

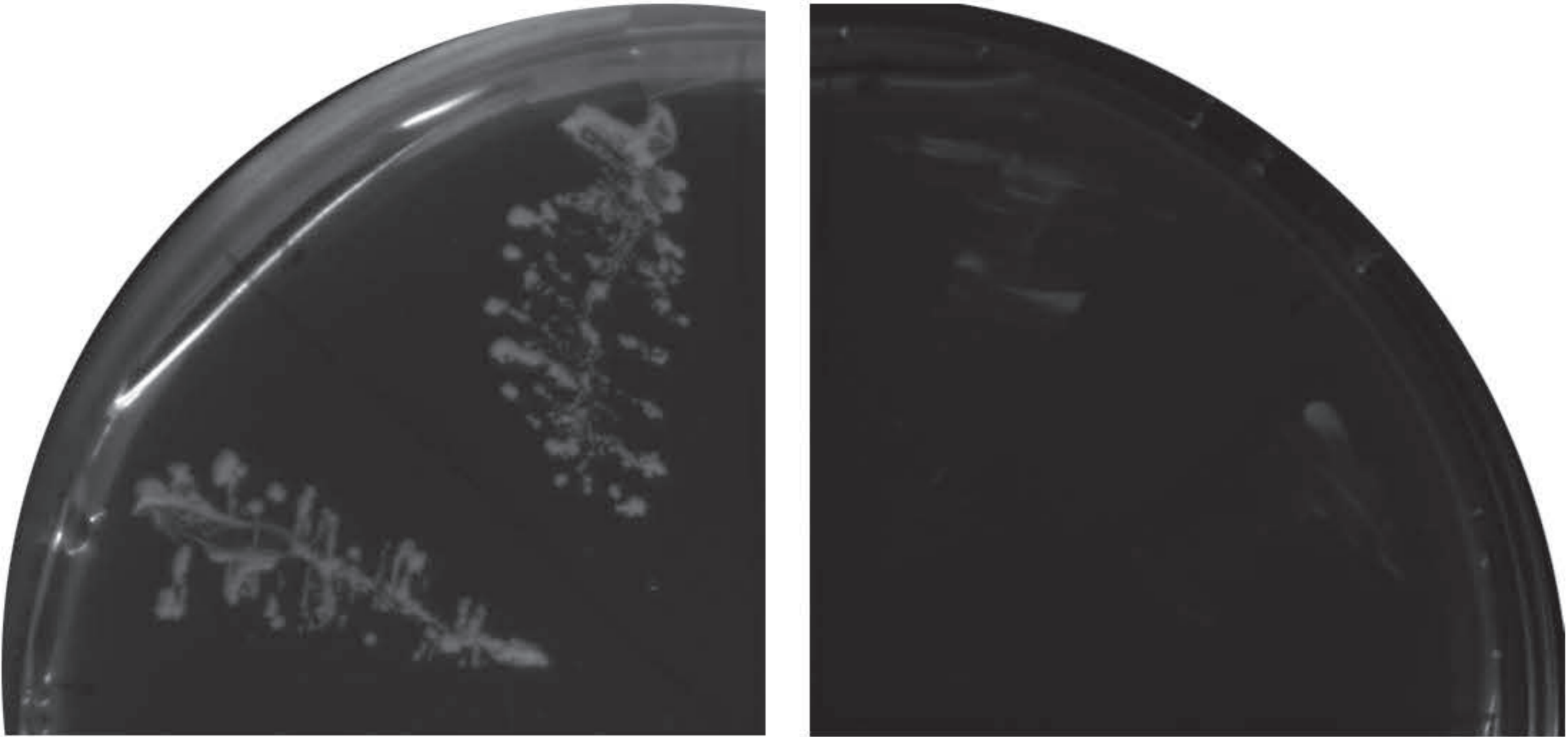

TEF2 EFL

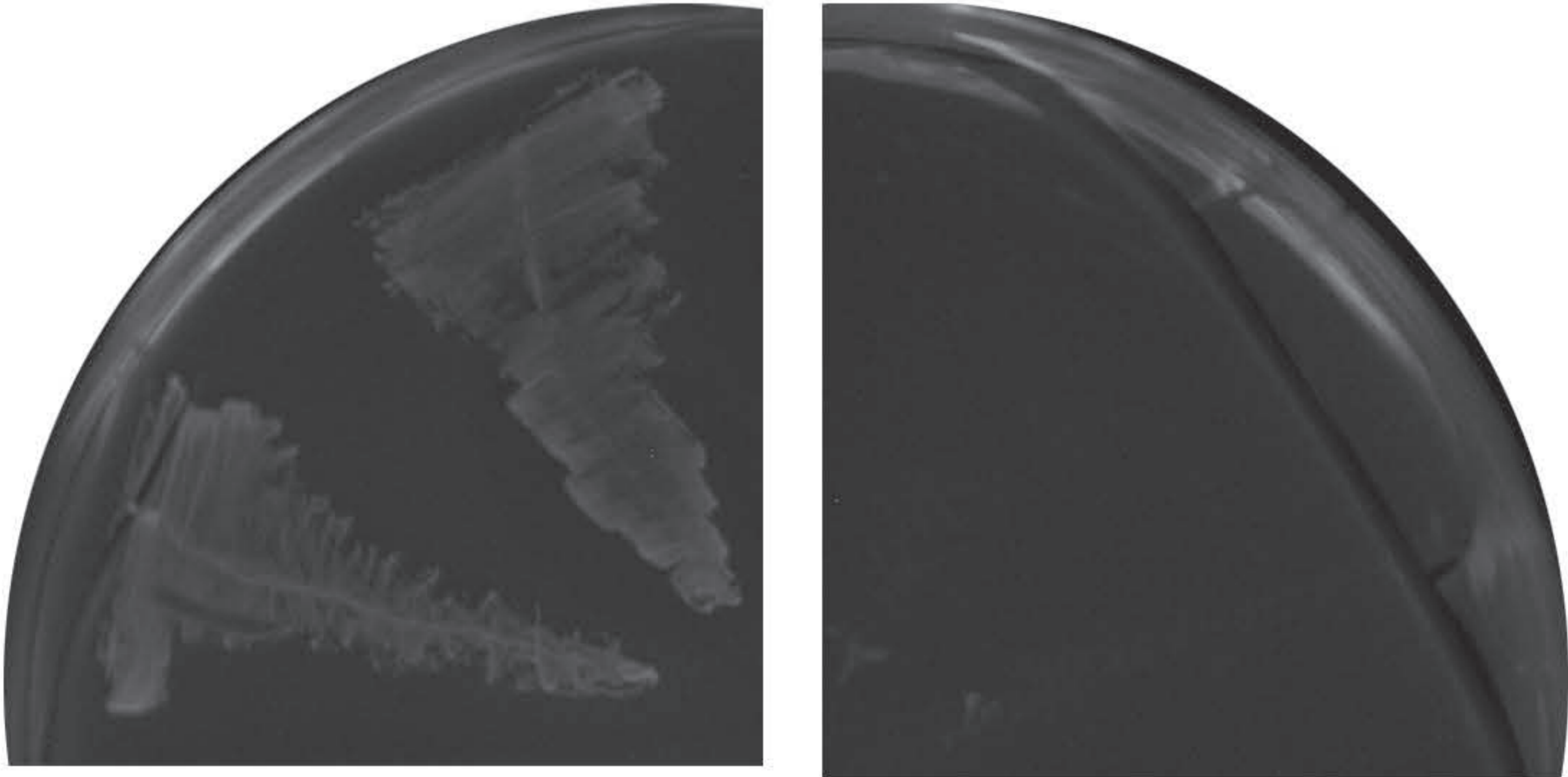

TEF5 EFL

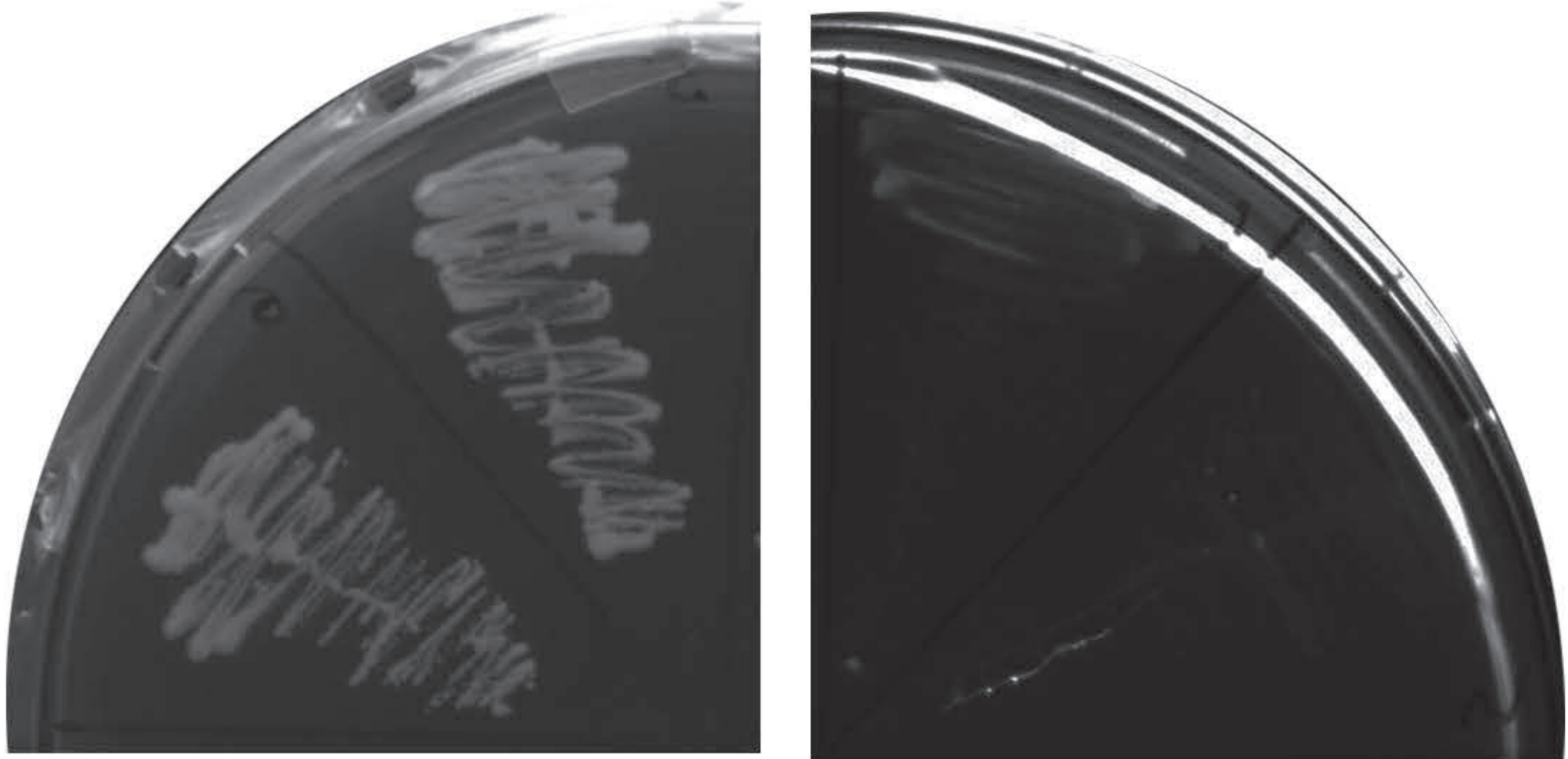

Supplement: Additional file 4: Figure S2 — Both S. cerevisiae double eEF1Bα and eEF1A knock-out and eEF1Bα single knock-out are not complemented in vivo by Monosiga brevicollis EFL. See Additional file 3 for legend. [file 1471-2148-14-35-S4.pdf]
