# Supplementary material for: An evolutionary ratchet leading to loss of elongation factors in eukaryotes
Source: BMC Evol Biol. 2014 Feb 24;14:35. doi: 10.1186/1471-2148-14-35 (PMC3938643; doi:10.1186/1471-2148-14-35)
Supplement: Additional file 1: Table S1 — Presence and absence of EFL eEF1A, eEF1Bα with sequence ID numbers. Sequences are ordered by taxonomy. Paler colours indicate that a sequence is present, but highly divergent. [file 1471-2148-14-35-S1.pdf]

Additional file 1 Table S1: Presence and absence of EFL eEF1A, eEF1B! with sequence ID numbers

Sequences are ordered by taxonomy. Paler colours indicate that a sequence is present, but highly divergent.

		Species	EFL	EF1A	EF1B	notes
Opisthokonta	Fungi	*Allomycis macrogynus*	AMAG_15142, AMAG_11300, AMAG_13011, AMAG_14104	AMAG_17705, AMAG_02240[frag]	-	
		Blastocladiella emersonii	gi|117422540|gb|ABK34893.1|, gi|82592726|gb|ABB84534.1|[frag]	-	-	
		Conidiobolus cornatus	EST:gi|21649885|gb|BQ622716.1|BQ622716	-	-	
		Mortierella verticillata	-	MVEG_01883	MVEG_03078, MVEG_05990	
		Saccharomyces cerevisiae	-	gi|6319594|ref|NP_009676.1|, gi|6325337|ref|NP_015405.1|	gi|68845631|sp|P32471.4|EF1B_YEAST	
		Spizellomycete punctatus	SPPG_08407	SPPG_0649	-	
		Aspergillus niger	-	gi|317037311|ref|XP_003188975.1|	gi|317030381|ref|XP_001392465.2|	
		Cryptococcus neoformans	-	gi|58262104|ref|XP_568462.1|	gi|58265036|ref|XP_569674.1|	
		Acidomyces richmondensis	-	jgi|Aciri1_iso|314428|CE136362_647183	jgi|Aciri1_iso|177148|estExt_fgenesh1_pg.C_5840002	
		Batrachochytrium dendrobatidis	-	jgi|Batde5|19314|estExt_Genewise1Plus.C_40375	jgi|Batde5|8958|e_gw1.1.1137.1	
		Acremonium alcalophilum	-	jgi|Acral2|2046471|estExt_Genemark1.C_2_t10432	jgi|Acral2|1060882|Acral1_c3a.fgenesh2_kg.2_#_254_#_Contig2391	
		Antonospora locustae (2493)	DR043853	-	-	
		Xanthoria parietina	-	jgi|Xanpa1|62882|gm1.2537_g	jgi|Xanpa1|70822|gm1.10477_g	
		Wolfiporia cocos	-	jgi|Wolco1|147680|gm1.625_g	jgi|Wolco1|25778|fgenesh1_kg.6_#_251_#_isotig10127	
		Phycomyces blakesleeanus	-	jgi|Phybl2|15575|Phybl1.e_gw1.2.294.1	jgi|Phybl2|35925|Phybl1.estExt_Genewise1Plus.C_20502	
		Rhizopus delemar	-	jgi|Rhior3|17239|RO3G_15350	jgi|Rhior3|539|RO3G_11503	
		Coemansia reversa	-	jgi|Coere1|9068|gm1.3777_g	jgi|Coere1|86978|fgenesh1_pg.14_#_127	
		Piromyces sp.E2	-	jgi|PirE2_1|20975|estExt_Genemark1.C_2440001	jgi|PirE2_1|24699|gw1.120.14.1	
		Mucor circinelloides	-	jgi|Mucci2|153876|fgenesh1_pm.07_#_133	jgi|Mucci2|153951|fgenesh1_pm.07_#_208	
		Wickerhamomyces anomalus	-	jgi|Wican1|66780|estExt_Genemark1.C_1_t20463	jgi|Wican1|101038|estExt_fgenesh1_pm.C_4_t10447	
	Ichthyosporea	*Amoebidium parasiticum*	-	EST: gi|109737174|gb|EC629577.1|EC629577	EST: gi|109736153|gb|EC628556.1|EC628556	
		Sphaeroforma arctica	SARC_1268	-	-	
	Filasterea	*Capsaspora owczarzaki*	-	CAOG_07807, CAOG_08980	CAOG_04931, CAOG_06302	
	Choanoflagellatea	*Monosiga brevicollis*	jgi|Monbr1|36952|estExt_fgenesh1_pg.C_90087	-	-	
		Monosiga sp. (ATCC 50635; *M. ovata*)	-	gi|53829544|gb|AAU94651.1|	gi|106850024|gb|EC168401.1|EC168401	
		Salpingoeca rosetta	PTSG_02256	-	-	
	Metazoa	*Homo sapiens*	-	gi|4503471|ref|NP_001393.1|, gi|4503475|ref|NP_001949.1|, gi|169162126|ref|XP_001130188.2|, gi|169162824|ref|XP_001131535.2|, gi|239752724|ref|XP_001715130.2|	gi|4503477|ref|NP_001950.1|	
		Drosophila melanogaster	-	gi|17137572|ref|NP_477375.1|, gi|24652838|ref|NP_725085.1|, gi|17864154|ref|NP_524611.1|, gi|24651721|ref|NP_733449.1|, gi|45553807|ref|NP_996315.1|, gi|45553816|ref|NP_996316.1|	gi|13124189|sp|O96827.3|EF1B_DROME	
		Branchiostoma floridae	-	gi|261289491|ref|XP_002604722.1|, gi|261289495|ref|XP_002604724.1|, gi|260781721|ref|XP_002585950.1|, gi|260810141|ref|XP_002599862.1|, gi|260810133|ref|XP_002599858.1|, gi|260797930|ref|XP_002593953.1|, gi|260810137|ref|XP_002599860.1|, gi|260810145|ref|XP_002599864.1|	jgi|Brafl1|115322|estExt_fgenesh2_pm.C_2060009	
		Trichoplax adhearens	-	jgi|Triad1|36635|estExt_Genewise1.C_210042, jgi|Triad1|31868|e_gw1.21.103.1	jgi|Triad1|36550|estExt_Genewise1.C_150163, jgi|Triad1|38008|estExt_Genewise1Plus.C_70767[frag]	
		Capitella teleta	-	jgi|Capca1|148876|estExt_Genewise1.C_670028, jgi|Capca1|149613|estExt_Genewise1.C_26720010	jgi|Capca1|179520|estExt_Genewise1Plus.C_15490001, jgi|Capca1|155717|estExt_Genewise1.C_12220001_fusion, jgi|Capca1|228652|estExt_fgenesh1_pg.C_10060001	
		Lottia gigantica	-	jgi|Lotgi1|239271|estExt_fgenesh2_pg.C_sca_2180008	jgi|Lotgi1|193902|estExt_Genewise1.C_sca_550213	
		Hydra magnipapillata (146429)	-	gi|221124734|ref|XP_002160595.1|	gi|221123731|ref|XP_002162369.1|, gi|221127438|ref|XP_002160577.1|	
		Oscarella carmela (11176)		EST: gi|110573384|gb|EC374292.1|EC374292	EST: gi|110570870|gb|EC371778.1|EC371778	
		Pleurobrachia pileus (35290)	-	gi|294433601|gb|FP988813.1|FP988813	gi|294433120|gb|FP989537.1|FP989537	
		Nematostella vectensis	-	jgi|Nemve1|247215|estExt_fgenesh1_pg.C_2780044	jgi|Nemve1|219557|fgenesh1_pg.scaffold_420000007, jgi|Nemve1|173281|estExt_gwp.C_2430070, jgi|Nemve1|155941|e_gw.9414.2.1	
	Apusozoa	*Thecamonas trahens*	AMSG_04544, AMSG_04543, AMSG_07674, AMSG_04551	AMSG_03948	AMSG_01300	eEF1B apparent fusion with kinase
Amooebozoa	Amoebozoa	*Dictyostelium discoideum*	-	gi|66824969|ref|XP_645839.1|, gi|66825247|ref|XP_645978.1|	gi|66810023|ref|XP_638735.1|	
		Dictyostelium purpureum	-	gi|330792728|ref|XP_003284439.1|	gi|330794872|ref|XP_003285500.1|	
		Mastigamoeba balamuthi (20111)	-	gi|18055257|gb|BE636668.2|BE636668	gi|110022847|gb|EC708730.1|EC708730	
		Acanthamoeba castellanii (13784)	-	gi|106792843|gb|EC111220.1|EC111220, gi|106792859|gb|EC111236.1|EC111236	gi|106788924|gb|EC107301.1|EC107301	
		Hartmannella vermiformis (9553)	-	gi|106813686|gb|EC132063.1|EC132063	gi|106813142|gb|EC131519.1|EC131519	
		Physarum polycephalum (25393)	-	gi|110074353|gb|EC760236.1|EC760236, gi|110074278|gb|EC760161.1|EC760161	gi|110070806|gb|EC756689.1|EC756689, gi|110068440|gb|EC754323.1|EC754323	
Cryptophyta	Cryptophyta	*Guillardia theta*	jgi|Guith1|149923|fgenesh2_kg.2_#_103_#_1003_1_CCFI_CCFN_EXTA_EXTB	jgi|Guith1|84077|estExt_Genewise1Plus.C_30325	jgi|Guith1|154358|fgenesh2_kg.73	
		Rhodomonas salina	EST:gi|117417090|gb|EG749683.1|EG749683	-	-	
Excavata	Kinetoplastida	*Trypanosoma brucei*	-	gi|71746820|ref|XP_822465.1|, gi|71746822|ref|XP_822466.1|, gi|71746818|ref|XP_822464.1|	gi|71747546|ref|XP_822828.1|, gi|72388166|ref|XP_844507.1|, gi|72388170|ref|XP_844509.1|	
		Bodo saltans	-	BS46520	BS19175	(GeneDB)
		Leishmania braziliensis	-	gi|154335074|ref|XP_001563777.1|	gi|154345682|ref|XP_001568778.1|, gi|154336255|ref|XP_001564363.1|, gi|154336251|ref|XP_001564361.1|, gi|154336263|ref|XP_001564367.1|	
	Euglenozoa	*Diplonema papillatum*	gi|226347415|gb|ACO50118.1|	-	-	checked with ESTs
	Heterolobosea	*Naegleria gruberi*	-	jgi|Naegr1|29541|e_gw1.2.255.1	jgi|Naegr1|71637|fgeneshNG_pg.scaffold_48000024	
	Diplomonada	*Giardia lamblia*	-	gi|159108518|ref|XP_001704529.1|, gi|159108554|ref|XP_001704547.1|	gi|159107582|ref|XP_001704069.1|	
Haptophyceae	Haptophyceae	*Isochrysis galbana*	EST:gi|106828296|gb|EC146673.1|EC146673	-	-	
		Emiliania huxleyi	jgi|Emihu1|462457|estExtDG_fgeneshEH_pg.C_140136, jgi|Emihu1|78778|e_gw1.8515.1.1	-	-	
		Pavlova lutheri	EST: gi|106859211|gb|EC177588.1|EC177588		-	
Stramenopiles	Heterokontophyta	*Thalassiosira pseudonana*	gi|224007705|ref|XP_002292812.1|	gi|224015598|ref|XP_002297450.1|, gi|223998706|ref|XP_002289026.1|	-	
		Phaeodactylum tricornutum	-	gi|219113439|ref|XP_002186303.1|, gi|219122524|ref|XP_002181593.1|	jgi|Phatr2|48822|estExt_fgenesh1_pg.C_chr_190140	
		Pseudo-nitzschia multiseries	jgi|Psemu1|300345|fgenesh1_kg.10	jgi|Psemu1|321099|estExt_fgenesh1_pm.C_12740003	-	
		Aureococcus anophagefferens	-	jgi|Auran1|36925, jgi|Auran1|36932	jgi|Auran1|61384|fgenesh2_pg.C_scaffold_2000392	could only be found with tblastn
		Fragilariopsis cylindrus	jgi|Fracy1|269944|estExt_fgenesh2_kg.C_90222, jgi|Fracy1|270244|estExt_fgenesh2_kg.C_110014	jgi|Fracy1|250437|fgenesh2_pg.31_#_36	-	
	Oomycetes	*Phytophthora ramorum*	-	jgi|Phyra1_1|71257|fgenesh1_pm.C_scaffold_10000010, jgi|Phyra1_1|71250|fgenesh1_pm.C_scaffold_10000003	jgi|Phyra1_1|71498|fgenesh1_pm.C_scaffold_23000008, jgi|Phyra1_1|45783|gwEuk.26.15.1[frag]	
		Phytophthora capsici	-	jgi|Phyca11|510859|fgenesh2_kg.PHYCAscaffold_70_#_5_#_Contig3.1, jgi|Phyca11|504383|fgenesh2_kg.PHYCAscaffold_7_#_135_#_4098508:8	jgi|Phyca11|43072|gw1.28.98.1, jgi|Phyca11|504578|fgenesh2_kg.PHYCAscaffold_8_#_147_#_4101647:4	
		Pythium oligandrum	EST: gi|166296477|gb|EV245795.1|EV245795	-	-	
		Pythium ultimum	EST: gi|148601142|gb|EL774728.2|EL774728	-	Short nonsense transcript:gi|207393250|gb|FE988442.1|FE988442	
		Phytophthora sojae	-	jgi|Physo3|354065|estExt_fgenesh1_kg.C_2_t10417, jgi|Physo3|354062|estExt_fgenesh1_kg.C_2_t10410	jgi|Physo3|354390|estExt_fgenesh1_kg.C_3_t10382, jgi|Physo3|353349|estExt_fgenesh1_kg.C_1_t10042	
Alveolata	Dinophyceae	*Karenia brevis* (65266)	EST: gi|159004894|gb|EX961328.1|EX961328	EST: gi|48705752|gb|CO063583.1|CO063583	-	
		Alexandrium catenella (9848)	EST: gi|186959837|gb|EX460213.1|EX460213	-	-	
		Alexandrium ostenfeldii (10875)	EST: gi|307929265|gb|HO663348.1|HO663348	-	-	
		Amphidinium carterae	gi|193891003|gb|ACF28655.1|, gi|193891037|gb|ACF28672.1|	-	-	checked with ESTs
		Lingulodinium polyedrum (74974 nt + 3640 EST)	ESTs: gi|39542136|gb|CD810812.1|CD810812, gi|39541175|gb|CD809851.1|CD809851	-	-	
		Karlodinium micrum	gi|110225900|gb|ABG56231.1|, gi|157093189|gb|ABV22249.1|	-	-	checked with ESTs
		Symbiodinium sp.CladeC	EST: gi|145312101|gb|EH058096.1|EH058096	EST: gi|145311420|gb|EH037774.1|EH037774	-	
		Heterocapsa triquetra	gi|157783495|gb|ABV72555.1|, gi|157783497|gb|ABV72556.1|, gi|157783499|gb|ABV72557.1|	-	-	checked with ESTs
		Oxyrrhis marina	gi|106073631|gb|ABF82158.1|	-	-	checked with ESTs
	Ciliophora	*Tetrahymena thermophila*	-	gi|118399778|ref|XP_001032213.1|, gi|118373835|ref|XP_001020110.1|, gi|118379857|ref|XP_001023094.1|, gi|118373371|ref|XP_001019879.1|, gi|118399772|ref|XP_001032210.1|, gi|146177363|ref|XP_001470896.1|	gi|118371716|ref|XP_001019056.1|, gi|118370671|ref|XP_001018536.1|	
		Paramecium tetraurelia	-	gi|145481817|ref|XP_001426931.1|, gi|145533444|ref|XP_001452467.1|, gi|145481305|ref|XP_001426675.1|, gi|145550331|ref|XP_001460844.1|, gi|145480491|ref|XP_001426268.1|, gi|145543404|ref|XP_001457388.1|, gi|145543408|ref|XP_001457390.1|	gi|145551432|ref|XP_001461393.1|, gi|145482843|ref|XP_001427444.1|	
	Perkinsea	*Perkinsus marinus*	gi|294874827|ref|XP_002767118.1|, gi|294874829|ref|XP_002767119.1|, gi|294936907|ref|XP_002781906.1|, gi|294951299|ref|XP_002786917.1|, gi|294929718|ref|XP_002779342.1|, gi|294929720|ref|XP_002779343.1|, gi|294889473|ref|XP_002772830.1|, gi|294889469|ref|XP_002772828.1|, gi|294865412|ref|XP_002764405.1|, gi|294954686|ref|XP_002788268.1|, gi|294867165|ref|XP_002764983.1|, gi|294943558|ref|XP_002783908.1|, gi|294879170|ref|XP_002768583.1|, gi|294942936|ref|XP_002783717.1|, gi|294875717|ref|XP_002767442.1|, gi|294879534|ref|XP_002768714.1|, gi|294866598|ref|XP_002764776.1|, gi|294934611|ref|XP_002781165.1|, gi|294871312|ref|XP_002765887.1|, gi|294939719|ref|XP_002782552.1|, gi|294951375|ref|XP_002786949.1|	-	-	
	Apicomplexa	*Toxoplasma gondii*	-	gi|237839941|ref|XP_002369268.1|, gi|237841903|ref|XP_002370249.1|	gi|237833993|ref|XP_002366294.1|, gi|237842761|ref|XP_002370678.1|	
		Babesia bovis	-	gi|156087152|ref|XP_001610983.1|, gi|156087154|ref|XP_001610984.1|	gi|156084035|ref|XP_001609501.1|	
		Plasmodium falciparum	-	gi|124513850|ref|XP_001350281.1|, gi|124513852|ref|XP_001350282.1|	gi|124505049|ref|XP_001351266.1|, gi|124506813|ref|XP_001352004.1|	
Rhizaria	Cercozoa	*Bigelowiella natans*	jgi|Bigna1|36951|e_gw1.17.41.1, jgi|Bigna1|48294|estExt_Genewise1.C_250046, jgi|Bigna1|73872|fgenesh1_pg.26_#_120	jgi|Bigna1|67808|fgenesh1_pg.4_#_302	-	
		Paracercomonas marina	-	EST: gi|156132164|gb|EW706280.1|EW706280	EST: gi|156130706|gb|EW704829.1|EW704829[frag]	fragment
	Foraminifera	*Reticulomyxa filosa*	gi|193875926|gb|ACF24599.1|	-	-	checked with ESTs
Viridiplantae	Streptophyta	*Selaginella moellendorffii*	-	gi|302770006|ref|XP_002968422.1|, gi|302770000|ref|XP_002968419.1|, gi|302774332|ref|XP_002970583.1|, gi|302774324|ref|XP_002970579.1|	gi|302818954|ref|XP_002991149.1|, gi|302775011|ref|XP_002970922.1|	
		Physcomitrella patens	-	gi|168000711|ref|XP_001753059.1|, gi|168000955|ref|XP_001753181.1|, gi|168000927|ref|XP_001753167.1|, gi|168027930|ref|XP_001766482.1|, gi|168021329|ref|XP_001763194.1|, gi|168021343|ref|XP_001763201.1|, gi|168021337|ref|XP_001763198.1|	gi|168061102|ref|XP_001782530.1|, gi|168039139|ref|XP_001772056.1|, gi|168006889|ref|XP_001756141.1|	
		Mesostigma viride	gi|88911222|gb|ABD58901.1|	-	-	checked with ESTs
	Chlorophyta	*Chlamydomonas reinhardtii*	gi|159476938|ref|XP_001696568.1|, gi|159488713|ref|XP_001702347.1|	gi|159471079|ref|XP_001693684.1|	-	
		Chlamydomonas incerta	gi|74272649|gb|ABA01120.1|	-	-	
		Ulva prolifera (6738)	ESTS: gi|334723922|gb|GW323267.1|GW323267, gi|334724038|gb|GW323383.1|GW323383, gi|334724087|gb|GW321937.1|GW321937, gi|334723273|gb|GW325204.1|GW325204	EST: gi|334723910|gb|GW323255.1|GW323255	-	
		Polytomella parva	EST: gi|110065228|gb|EC751111.1|EC751111	-	-	
		Ostreococcus tauri	jgi|Ostta4|23014|estExt_fgenesh1_pm.C_Chr_13.00010011, jgi|Ostta4|29385|estExt_gwp_GeneWisePlus.C_Chr_13.00010306	-	-	
		Ostreococcus sp.RCC809	jgi|OstRCC809_2|65043|fgenesh1_pg.chr_13_#_160, jgi|OstRCC809_2|95346|EuGene.1300010093	-	-	
		Ostreococcus lucimarinus	jgi|Ost9901_3|27060|eugene.1300010082, jgi|Ost9901_3|27198|eugene.1300010220, jgi|Ost9901_3|29711|eugene.2100010085	-	-	
		Prototheca wickerhamii	EST: gi|106864665|gb|EC183042.1|EC183042	-	-	
		Volvox carteri	jgi|Volca1|102756|estExt_fgenesh4_pg.C_10352, jgi|Volca1|103661|estExt_fgenesh4_pg.C_80129	jgi|Volca1|34197|gw1.42.36.1,jgi|Volca1|37858|gw1.42.53.1	-	
		Micromonas sp RCC299	jgi|MicpuN3|91047|estExt_Genewise2.C_Chr_060412, jgi|MicpuN3|91112|estExt_Genewise2.C_Chr_060641	-	-	
		Chlorella variabilis	gi|307108419|gb|EFN56659.1|, gi|307108632|gb|EFN56872.1|	-		
		Helicosporidium sp.	gi|54399516|gb|AAV34148.1|	-	-	checked with ESTs
		Scenedesmus obliquus	gi|224593223|gb|ACN59931.1|	-	-	
	Rhodophyta	*Cyanidioschyzon merolae*	-	gnl|CMER|CMH226C	gnl|CMER|CME120C	(C. merolae DB)
		Chondrus crispus	EST:gi|62995946|gb|CO652990.1|CO652990	-	-	
		Gracilaria tenuistipitata	EST: gi|327361628|gb|HS979457.1|HS979457	-	-	
		Gracilaria changii	EST: gi|120462771|gb|DV963156.1|DV963156 GC01129	-	-	
