# Supplementary material for: An evolutionary ratchet leading to loss of elongation factors in eukaryotes
Source: BMC Evol Biol. 2014 Feb 24;14:35. doi: 10.1186/1471-2148-14-35 (PMC3938643; doi:10.1186/1471-2148-14-35)
Supplement: Additional file 3: — Supplementary Methods, full legends for figures S1 and S2, and Table S2 of strains and plasmids used in the study. [file 1471-2148-14-35-S3.pdf]

Additional file 3

Supplementary Methods

Preparation of *Monosiga brevicollis* genomic DNA and sub-cloning of the EFL gene

A liquid culture of *Monosiga brevicollis* supplemented with *Enterobacter aerogenosa* as a food source was kindly provided by Dr. Barry Leadbeater, University of Birmingham. The mixed culture was grown on bacterial Petri dishes in 10 ml of synthetic seawater was supplemented with one grain of sterilized rice as bacterial food source. Synthetic seawater reconstituted from 38-40g per liter of synthetic seawater salt mix for tropical aquariums (Tropica Aquarium plants, Denmark) in milli-Q water, autoclaved and filtered, and with 40 µl of organic supplement (0.4 g of Difco pepton, 0.086 g of yeast extract per 100 ml milli-Q water, autoclaved and stored at 4°C).

Cultures were grown for 10-14 days at room temperature, then material from 10 confluent dishes was collected with a cell scraper and pelleted by centrifugation at 17 000 x g for 30 min at 4°C. Genomic DNA was prepared from the resulting cell mass as described in (King et al. 2009) using Quick-gDNA Miniprep kit (Zymo Research). The full-length *M. brevicollis* EFL gene (GenBank ID AY026073.1) was amplified from the genomic DNA by PCR using forward primer 5´ACCATGGTTACTGACGGCAAGCAAC3´ and reverse primer 5´AAAGCTAGCACGCTCGACCTTG3´, and sub-cloned into pTZ 57R/T vector (Instaclone PCR Cloning kit, Fermentas).

In vivo complementation of the eEF1A and eEF1Bα knock-out in *S. cerevisiae* by *M. brevicollis* EFL

For testing the complementation of yeast eEF1Bα (gene name TEF5) and eEF1A (encoded by two paralogs, TEF1 and TEF2) genomic deletion with *M. brevicolis* EFL we first cloned the EFL gene into a single copy centromere vector pRS317 with a *LYS2* selectable marker in the native genomic background of either TEF5, TEF1 or TEF2 ORFs.

The constructs were made using the yeast *in vivo* recombination technique (Oldenburg et al. 1997). The TEF5 ORF with approximately 400 bp up- and downstream flanking regions was PCR amplified from the plasmid JWB2937 (Kinzy, Woolford 1995) kindly provided by Terry G. Kinzy and cloned into the pRS317 vector at the ApaI and NotI sites resulting in plasmid pRS317_TEF5f. Both 500 bp flanks of TEF1 and TEF2 were PCR amplified from genomic DNA of W303 wild-type strain and cloned into pRS317 at PstI and NotI sites generating plasmids pRS317_TEF1f and pRS317_TEF2f, respectively. Then the ORFs in the vectors described above were replaced with the EFL ORF. To that end a wild type lysine-auxotrophic yeast strain DUL2 (courtesy of Thomas D Fox) was co-transformed with two linear DNA molecules, namely either pRS317_TEF5f, pRS317_TEF1f or pRS317_TEF2f linearized between flanking regions by using a appropriate unique restriction site and, second, the EFL ORF amplified by PCR with primers containing 40 bp 3´-proximal parts homologous to the 40 nucleotides before and after the ORF being replaced. The resulting constructs containing the EFL gene in the genetic background of TEF5 (pRS317_TEF5f_EFL), TEF1 (pRS317_TEF1f_EFL) or TEF2 (pRS317_TEF2f_EFL) were purified by a modified procedure using a miniprep plasmid purification kit (QIAGEN) (http://labs.fhcrc.org/simon/ystmini.pdf).

S. cerevisiae strains TKY961 and JWY4231 kindly provided by Terry G. Kinzy were used for *in vivo* complementation of eEF1A and eEF2Bα knock-outs by EFL. In the TKY961 strain, knock-out of both ORFs coding for eEF1A (TEF1 & TEF2) as well as of TEF5 gene encoding eEF1Bα are complemented by a *URA3*-containing plasmid carrying a mutant variant of eEF1A (R164K) that can support yeast viability without the guanidine exchange factor eEF1B (Ozturk, Kinzy 2008). In JWY4231, the TEF5 deletion is complemented by

expression of the wild type TEF5 gene from a ura3-containing plasmid. TKY961 was transformed with either pRS317_TEF1f_EFL or pRS317_TEF2f_EFL, and JWY4231 was transformed with pRS317_TEF1f_EFL or pRS317_TEF5f_EFL.

Plating the *S. cerevisiae* strains onto selective media containing 5-fluoroorotic acid (5-FoA) induced eviction of the plasmid-encoded TEF1, TEF2 or TEF5, and the efficiency of eEF1A and eEF1Bα or just eEF1Bα complementation by EFL was determined by assessing the yeast growth after five days of incubation at 30°C.

All strains and plasmids used throughout this study are listed in supplementary table 1.

Supplementary figure legends:

Supplementary Fig S1: Phylogenies of eEF1A and EFL
Maximum likelihood phylogenies of A) eEF1A and B) EFL are shown with branch support values indicated where support is greater than 50% Maximum Likelihood bootstrap percentage (MLBP). Support is indicated in the format MLBP/BIPP (Bayesian inference posterior probability). Stars following taxon names indicate those organisms that also carry intact or degraded EFL, eEF1A or eEF1Bα. Grey taxa names show those sequences are degrading in primary sequence, but still maintain enough sequence similarity to be included in phylogenetic analysis. A paralog of EFL in *Bigelowiella natans* that is nuclear-encoded, but periplastid-targeted (Gile, Keeling 2008) is indicated. The scale bar at the bottom of each tree shows the relation of branch length to number of substitutions per site.

Supplementary Fig S2: Both *S. cerevisiae* double eEF1Bα and eEF1A knock-out and eEF1Bα single knock-out are not complemented *in vivo* by *Monosiga brevicollis* EFL
S. cerevisiae strain TKY961 was transformed by the plasmid expressing *M. brevicollis* EFL gene under the control of the ~400 nt flanking regions of eEF1A-encoding gene TEF1 (**A**, upper panel) or eEF1A-encoding gene TEF2 (**A**, lower panel), and strain JWY4231 was transformed by the plasmid expressing EFL gene in the genomic context of eEF1Bα-encoding gene TEF5 (**B**, upper panel) or eEF1A-encoding gene TEF1 (**B**, lower panel). The resulting strains were grown on full synthetic medium (left segment of the plate) or on the medium containing 5-fluoroorotic acid (right segment of the plate) for five days. Two representative clones of each strain are shown. In the presence of 5-FoA ura3-containing plasmids pTEF1 URA3(R164K) in TKY961 and pTEF5 URA3 in JWY4231 are expelled, resulting in the loss of either both eEF1Bα and eEF1A or just eEF1Bα, respectively. The absence of yeast growth indicates absence of complementation.

Supplementary table S2: *S. cerevisiae* strains and plasmids used in the study

Strain or plasmid	Genotype	Reference
TKY961	MATα ura3-52 leu2-3,112 trp1Δ1 lys2-20 met2-1his4-713 tef1::LEU2 tef2Δ tef5::TRP1 pTEF1_URA3(R164K)	(Ozturk et al. 2006)
JWY4231	MATα ura3-52 trp1Δ101 lys2-801 leu2Δ1 met2-1 his4-713 tef5::TRP1 pTEF5_URA3	(Kinzy, Woolford 1995)
DUL2	D273-10B MATa lys2 ura3	Provided by Thomas D Fox
pRS317_TEF1f_EFL	pRS317 centromere shuttle vector with the *M. brevicolis* EFL ORF cloned under native flanking regions of TEF1	This study
pRS317_TEF2f_EFL	pRS317 centromere shuttle vector with the *M. brevicolis* EFL ORF cloned under native flanking regions of TEF2	This study
pRS317_TEF5f_EFL	pRS317 centromere shuttle vector with the *M. brevicolis* EFL ORF cloned under native flanking regions of TEF5	This study
pJWB2937	TEF5 ORF cloned in YCp50 plasmid	(Kinzy, Woolford 1995)

Gile, GH, PJ Keeling. 2008. Nucleus-encoded periplastid-targeted EFL in chlorarachniophytes. Mol Biol Evol 25:1967-1977.

King, N, SL Young, M Abedin, M Carr, BS Leadbeater. 2009. Preparation of high-molecular-weight genomic DNA from Monosiga brevicollis and other choanoflagellates. Cold Spring Harb Protoc 2009:pdb prot5153.

Kinzy, TG, JL Woolford, Jr. 1995. Increased expression of Saccharomyces cerevisiae translation elongation factor 1 alpha bypasses the lethality of a TEF5 null allele encoding elongation factor 1 beta. Genetics 141:481-489.

Oldenburg, KR, KT Vo, S Michaelis, C Paddon. 1997. Recombination-mediated PCR-directed plasmid construction in vivo in yeast. Nucleic Acids Res 25:451-452.

Ozturk, SB, TG Kinzy. 2008. Guanine nucleotide exchange factor independence of the G-protein eEF1A through novel mutant forms and biochemical properties. J Biol Chem 283:23244-23253.

Ozturk, SB, MR Vishnu, O Olarewaju, LM Starita, DC Masison, TG Kinzy. 2006. Unique classes of mutations in the Saccharomyces cerevisiae G-protein translation elongation factor 1A suppress the requirement for guanine nucleotide exchange. Genetics 174:651-663.
